# Diagnostic value of antibodies against a modified citrullinated vimentin in rheumatoid arthritis

**DOI:** 10.1186/ar2008

**Published:** 2006-07-19

**Authors:** Christian Dejaco, Werner Klotz, Heike Larcher, Christina Duftner, Michael Schirmer, Manfred Herold

**Affiliations:** 1 Clinical Department of Internal Medicine, Division of General Internal Medicine, Innsbruck Medical University, Anichstrasse 35, 6020 Innsbruck, Austria; 2 General Hospital of the Elizabethenians, Voelkermarkterstrasse 15-19, 9020 Klagenfurt, Austria

## Abstract

Antibodies directed against citrullinated vimentin are members of the family of autoantibodies reactive with citrullinated proteins and are among the most specific serological markers for the diagnosis of rheumatoid arthritis (RA). This study was performed to test the diagnostic value of a newly developed enzyme-linked immunosorbent assay (ELISA) for the detection of antibodies against a genetically modified citrullinated vimentin (anti-MCV) in comparison with a second-generation anti-cyclic citrullinated peptides (anti-CCP2) ELISA test system. Blinded sera from 631 patients (409 consecutive out-patients and 222 randomly selected stored sera) with RA (*n *= 164) and non-RA (osteoarthritis [*n *= 120], polymyalgia rheumatica/giant cell arteritis [*n *= 80], spondyloarthritis [*n *= 36], and other inflammatory rheumatic or non-inflammatory disease [*n *= 67]) were tested for the presence of anti-MCV and anti-CCP2 antibodies according to the manufacturers' instructions. The diagnostic performance of the anti-MCV was comparable with the anti-CCP2 assay for the diagnosis of RA according to the calculated area under the curve (0.824; 95% confidence interval (CI) 0.778–0.870 versus 0.818; 95% CI 0.767–0.869) as analysed by receiving operating characteristic curve. When categorised with a cutoff value of 20.0 U/ml (as recommended by the manufacturer), sensitivity and specificity of the anti-MCV ELISA were 69.5% (95% CI 61.9%–76.5%) and 90.8% (86.9%–93.8%), respectively, compared with 70.1% (62.5%–77.0%) and 98.7% (96.7%–99.6%) of the anti-CCP2 assay. Using the cutoff values of 19.0 U/ml and 81.5 U/ml for the anti-MCV test to obtain a sensitivity and specificity identical to the anti-CCP2 assay, showed a reduced specificity (89.8%; 85.8%–92.9%) and sensitivity (53.7%; 45.7%–61.5%), respectively, of the anti-MCV ELISA compared with the anti-CCP2 test. In conclusion, the serum ELISA testing for anti-MCV antibodies as well as the anti-CCP-2 assay perform comparably well in the diagnosis of RA. In the high-specificity range, however, the anti-CCP2 assay appears to be superior to the anti-MCV test.

## Introduction

Rheumatoid arthritis (RA) is the most common inflammatory joint disease, with a prevalence between 0.5% and 1% worldwide [1]. In most patients, diagnosis of RA is based on the criteria proposed by the American College of Rheumatology (ACR) in 1987 consisting of clinical symptoms and radiological findings, whereas the only laboratory test included is the serum rheumatoid factor (RF) determination [2]. The ACR criteria, however, were primarily developed as classification criteria in established disease, and shortcomings in RA patients with recent-onset disease have now become evident [3]. Currently available data suggest that the diagnosis of RA can benefit from testing for antibodies to citrulline-containing peptides such as antiperinuclear factors (APFs), antifillagrin antibodies, antikeratin antibodies (AKAs), and anti-cyclic citrullinated peptides (anti-CCPs) [4-7]. Due to practical inconvenience, APF was never introduced into clinical routine, whereas detection of AKA by indirect immunofluorescence was among the main laboratory tests used before anti-CCP enzyme-linked immunosorbent assay (ELISA) kits became commercially available. The anti-CCP ELISA is based on highly purified synthetic peptides from dedicated libraries containing modified arginine residues (citrulline) serving as antigens, has a specificity comparable with AKA, and is more specific than APF and RF testing [8-10].

Historically, anti-Sa antibodies were first identified in a French Canadian patient whose name began with Sa. The reactivity of these antibodies was found to be highly specific for RA [11]. Subsequent studies confirmed the high degree of RA specificity, which exceeds 95%, in several populations tested [12-15]. The sensitivity of this antibody varied with the stage of the disease tested, ranging from 20%–25% in early RA cohorts to 47% in patients with more established disease [14,15]. The Sa antigen, originally derived from placental tissue, has recently been identified as citrullinated forms of vimentin [11,16]. Vimentin is an intermediate filament that is widely expressed in mesenchymal cells and macrophages and is easily detectable in synovium and fibroblast-like synoviocytes [17-19]. *In vivo*, vimentin is usually not in a citrullinated state, but deimination of this protein occurs in macrophages undergoing apoptosis. Anti-citrullinated vimentin antibodies may then emerge as a consequence of inadequate clearance of apoptotic material in patients with RA [20].

In this study, we tested the value of a newly developed ELISA for the detection of antibodies against a genetically modified citrullinated vimentin (anti-MCV) in comparison with an anti-CCP2-based ELISA system for the diagnosis of RA.

## Materials and methods

### Patients

Consecutive sera (*n *= 409) were obtained between October 2005 and February 2006 from patients visiting the rheumatic outpatient clinic (Clinical Department of Internal Medicine) of the Innsbruck Medical University (Innsbruck, Austria) and stored until final use. Frozen sera (*n *= 222) from patients with known inflammatory rheumatic diseases obtained between 2003 and 2005 were randomly selected and included in the analysis. The final diagnosis was used as the reference standard and was obtained by chart review. Unclear cases were discussed by three investigators (C Dejaco, C Duftner, and MH) and excluded if no definite diagnosis could be reached (Additional File 1). One hundred and sixty-four patients were diagnosed as having RA that met at least four out of the seven criteria according to the 1987 ACR classification [2]. In the rest of the cases, the following diagnoses were made: osteoarthritis (OA [*n *= 120]), polymyalgia rheumatica and/or giant cell arteritis (PMR/GCA [*n *= 80]) [21], spondyloarthritis (SpA [*n *= 36], including psoriatic arthritis [*n *= 10] [22], ankylosing spondylitis [*n *= 9] [23], and colitis-associated/undifferentiated SpA [*n *= 17]) [24], or other inflammatory rheumatic or non-inflammatory disease (*n *= 67; connective tissue disease [*n *= 12], crystal arthopathy [*n *= 4], fibromyalgia [*n *= 9], mechanical back pain or enthesopathy [*n *= 29], virus infection-associated arthralgia [*n *= 6], and myelodysplastic syndrome [*n *= 7]). Demographic, clinical, and serological data of patients with RA were obtained by chart review (Table 1). Also, data on the serum levels of immunoglobulin (Ig) M-RF (IgM-RF), which were routinely determined by a nephelometric method (normal values 0–13 U/ml), were retrieved by chart review. The mean ages of patients with RA and patients with non-RA were 60.4 (± 12.0) and 58.5 (± 15.7) years (not significant), respectively. In the RA group, there were slightly more women (85.4%) than in the control group (76.2%; *p *= 0.026 according to the χ^2 ^test with Yates correction). Patients with RA were under current treatment with single or combined disease-modifying anti-rheumatic drugs (methotrexate [*n *= 95], leflunomide [*n *= 21], azathioprine [*n *= 3], sulfasalazine [*n *= 13], hydroxychloroquine [*n *= 13], cyclosporine A [*n *= 2], and *Escherichia coli *extract OM-89 [*n *= 8]) alone or in combination with corticosteroids (*n *= 84), and tumour necrosis factor-α blocking agents (infliximab [*n *= 2], etanercept [*n *= 6], adalimumab [*n *= 10], or anakinra [*n *= 2]). No association was found between disease duration and markers of disease activity (data not shown). The study was approved by the local ethics committee. Written and informed consent was obtained from each patient.

### Anti-CCP2 and anti-MCV testing

Anti-MCV and anti-CCP2 testing was performed in an investigator-blinded fashion. Anti-CCP2 antibody reactivity was tested using a commercially available automated ELISA (EliA™ CCP Assay;Phadia GmbH, Freiburg, Germany) on a ImmunoCAP100 automatic analyzer (Phadia AB, Uppsala, Sweden) according to the manufacturer's recommendations. Values of 10.0 U/ml or greater were considered to be positive. Anti-MCV antibodies were measured using a recently launched and now commercially available ELISA (kindly provided by ORGENTEC Diagnostica GmbH, Mainz, Germany) according to the manufacturer's instructions [25]. In brief, serum samples were diluted 1:100 and incubated on MCV-coated microtiter wells for 30 minutes at room temperature on a horizontal shaking platform (100/second). Plates were washed three times and incubated with peroxidase-labeled anti-human IgG-conjugate for 15 minutes. 3,3',5,5'-Tetramethylbenzidine substrate was incubated for 15 minutes after additional washing. Color development was stopped with 1 M HCl solution, and the optical density of each well was measured with an Anthos 2020 microwell photometer (Anthos Labtec Instruments GmbH, Wals, Austria). Results are expressed in U/ml using a simple point-to-point curve-fitting method. Values of 20.0 U/ml or greater were considered to be abnormal according to manufacturer's recommendations. This cutoff was determined in a previous study of 232 healthy blood donors performed by ORGENTEC Diagnostica GmbH and comprises two standard deviations (SDs) of the mean anti-MCV titer in this cohort (according to the user manual of the anti-MCV ELISA provided by ORGENTEC Diagnostica GmbH and personal communication).

### Statistical analysis

Distributions of laboratory test results are described as the percentage per category for qualitative items and as mean (± SD) or median (and range) for quantitative items as stated. For anti-CCP2 and anti-MCV, the receiving operating characteristic (ROC) curve was constructed by plotting sensitivity against one minus specificity (1 – specificity), varying the cutoffs [9,26]. Diagnostic values of anti-MCV, anti-CCP2, and IgM-RF are described as sensitivity and specificity with 95% confidence interval (CI). To compare quantitative data, the Mann-Whitney *U *and the Kruskal-Wallis tests (for multiple comparisons) were performed using the SPSS program, version 11.0 (SPSS Inc., Chicago, IL, USA).

## Results

### Patient characteristics

Sera from 631 patients were included in the study. All sera were subjected to anti-MCV and anti-CCP testing. Sera from 170 patients (26.9%) were anti-MCV-positive (using the recommended cutoff value of 20.0 U/ml), whereas those from 137 (21.7%) tested positive in the anti-CCP2 assay (recommended cutoff value of 10.0 U/ml). Anti-MCV and anti-CCP2 were significantly, but not perfectly, correlated with each other (correlation coefficient = 0.594; *p *< 0.001 using Spearman's rank correlation coefficient). In 467 patients, a definite clinical diagnosis (164 patients with RA and 303 with non-RA) was available, and thus these diagnoses were included in the final analysis (Additional File 1). Data on IgM-RF were available in 415 (88.9%) out of these 467 patients, and 178 (42.9%) were positive for IgM-RF.

### Diagnostic value of anti-MCV and anti-CCP2 testing

Patients with RA had higher serum titers of anti-MCV antibodies (median 101.0 U/ml; range 2–1,094) than patients with OA (7.0 U/ml; 1–87), PMR/GCA (6.9 U/ml; 2–101), SpA (7.0 U/ml; 2–39), and other inflammatory rheumatic or non-inflammatory diseases (8.0 U/ml; 2–101; *p *< 0.001 for each comparison according to the Mann-Whitney *U *test).

**Figure 1:**
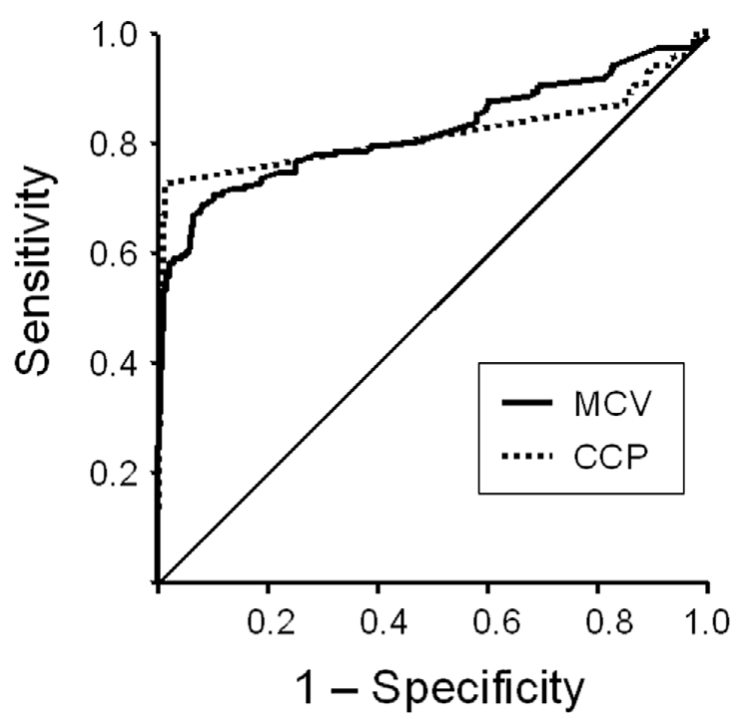
Comparison of the diagnostic values of the anti-modified citrullinated vimentin (MCV) and the anti-cyclic citrullinated peptide (CCP) 2 assay. Receiver operating curves of the anti-MCV and (second-generation) anti-CCP antibodies are shown. The sensitivity of each test is plotted against one minus specificity for varying cutoffs (values lower than the cutoff were considered negative, and other values were considered positive) (*n *= 467).

For direct comparison of the diagnostic values of the anti-MCV and the anti-CCP2 assays, we performed an ROC analysis. The calculated area under the curve (AUC) was comparable between the anti-MCV (0.824; 95% CI 0.778–0.870) and the anti-CCP2 ELISA (0.818; 0.767–0.869) (Figure 1).

For comparing the sensitivity and specificity of the two tests, we used not only the recommended cutoff value of 20.0 U/ml for the anti-MCV ELISA, but also two additional cutoffs, namely 19.0 and 81.5 U/ml. The results are summarised in Table 2. At the cutoff of 19.0 U/ml, both tests had an identical sensitivity, but the specificity of the anti-MCV was reduced. In contrast, when a cutoff of 81.5 U/ml was used in order to obtain an equal specificity for both assays, the anti-MCV ELISA demonstrated lower sensitivity than the anti-CCP2 test.

With respect to RA patients with recent onset of disease (<1 year; *n *= 23), the sensitivity of the anti-MCV (cutoff 20.0 U/ml) and anti-CCP2 assays were both lower (62.5%; 40.6%–81.2%) when compared with RA patients with established disease.

The diagnostic performances of the anti-MCV and the anti-CCP2 tests were similar in the consecutive cohort that included 49 patients with RA (29.9% out of all patients with RA) and 223 patients with non-RA (73.6% out of all patients with non-RA) compared with the total cohort, with a calculated AUC of 0.809 (0.725–0.893) for the anti-MCV ELISA and 0.843 (0.765–0.922) for the anti-CCP2 test. With the recommended cutoff values of 20.0 U/ml for the anti-MCV test and 10.0 U/ml for the CCP2 ELISA, both assays showed a sensitivity of 65.3% (50.4%–78.3%) to detect patients with RA; however, the specificity of the anti-MCV test (91.5%; 87.0%–94.8%) was lower than that of the anti-CCP2 ELISA (98.7%; 96.1%–99.7%). Taking the cutoff value of 81.5 U/ml at which the anti-MCV and the anti-CCP2 assays had identical specificities revealed the former assay to have lower sensitivity (49.0%; 34.4%–63.7%) in comparison with the latter.

Sensitivity and specificity of IgM-RF testing for the diagnosis of RA was 76.1% (68.6%–82.5%) and 77.7% (72.1%–82.7%), respectively, whereas in patients with early disease, IgM-RF revealed a sensitivity of 68.2% (45.1%–86.1%).

## Discussion

There is growing evidence that therapeutic intervention early in the course of RA leads to more efficient disease control, less joint damage, and better prognosis of disease outcome. Therefore, specific laboratory tests are desirable to help in the early differentiation of RA and other forms of rheumatic joint and connective tissue disease [14,27-29]. To date, a number of reports have demonstrated the high diagnostic value of antibodies directed against citrullinated proteins in the diagnosis of RA. Although the specificity was more than 90% in most studies, the sensitivity of the same antibodies varied between 33% and 87.2%, possibly reflecting diverse genetic backgrounds and/or methodological differences in diverse antigen preparations and detection techniques applied [30]. The diagnostic value of the anti-CCP2 test in this report was within the range of these earlier publications [7].

Anti-citrullinated vimentin antibodies have previously been detected in 23%–43% of patients with RA but in less than 8% of patients with non-RA and healthy controls by immunoblotting [11,13,15]. The objective of this study was to investigate the diagnostic accuracy of a novel and commercially available ELISA system for the detection of antibodies against a modified citrullinated vimentin (anti-MCV) in a large cohort of patients with confirmed diagnoses of RA and non-RA in direct comparison with the anti-CCP2 assay. As shown in Figure 1, the ROC analysis revealed a comparable overall diagnostic performance of the anti-MCV and anti-CCP2 ELISAs (AUCs of 0.824 and 0.818, respectively). However, the curves of both tests cross each other, and in the high-specificity range, which is clinically of greatest interest, the anti-CCP2 assay is the more sensitive test, whereas in the low-specificity range, the anti-MCV ELISA has a higher sensitivity. Indeed, taking the cutoff value of 81.5 U/ml to obtain identical specificity values for both test systems, the sensitivity of the anti-MCV assay (53.7%; 45.7%–61.5%) was significantly reduced (as the 95% CI did not include the corresponding sensitivity of the anti-CCP2 test). Using the cutoff of 19.0 U/ml with identical sensitivities of both assays resulted in a lower specificity of the anti-MCV test (89.8%; 85.8%–92.9%) compared with results from previous reports of anti-citrullinated vimentin and the anti-CCP2 assay [31]. Because a consecutive cohort of patients best reflects the number of patients with RA and non-RA seen in clinical routine, we analysed the performance of the anti-MCV test in this group separately. However, the diagnostic performance of the anti-MCV and anti-CCP2 ELISAs in this consecutive group did not differ from that in the entire cohort.

In patients with early course of disease, the sensitivity of the anti-MCV ELISA was lower at the recommended cutoff of 20.0 U/ml compared with those with long-standing RA but was equivalent to that of the anti-CCP2 test, detecting 62.5% of patients with RA. Previous studies reported anti-CCP antibodies in 47%–63% of patients with recent-onset RA, whereas anti-citrullinated vimentin antibodies have been reported in only one fifth of RA patients with early disease by immunoblotting [13,15], thus indicating a superior performance of the anti-MCV ELISA in this early-disease group compared with the immunoblotting technique [8,9,26,32]. Besides, the presence of anti-CCP antibodies has been shown to precede clinical onset of RA, and one might speculate that anti-MCV antibodies are also present in pre-disease serum samples [33]. Therefore, we cannot exclude the possibility that some of our anti-MCV (high)-positive patients without RA might develop RA in the future. In that case, the true degree of specificity of anti-MCV testing in this cohort might be underestimated [1].

The major limitations of our study are the small number of RA patients with early disease (*n *= 23) and the lack of follow-up data of the patients with non-RA. Prospective studies addressing the course of patients with undifferentiated early arthritis and long-term follow-up of the anti-MCV-positive patients with non-RA are necessary to evaluate the prognostic and diagnostic value of this test for patients with early RA. Because chart reviews partially lacked data of acute-phase reactants and clinical assessments, we did not investigate possible associations of the anti-MCV titer and other laboratory parameters and the clinical disease activity. Also, comparisons of the diagnostic value of anti-MCV with IgM-RF should be interpreted with caution due to the retrospective and incomplete retrieval of the IgM-RF. The sensitivity and specificity of IgM-RF in our present study are nevertheless consistent with published data [7].

## Conclusion

Autoantibodies to citrullinated proteins are specific for the diagnosis of RA. The anti-MCV ELISA is a novel, commercially available test system for the detection of antibodies directed against a modified citrullinated vimentin that demonstrates comparable overall diagnostic performance in relation to the anti-CCP2 assay according to the ROC analysis, with the calculated AUCs of 0.824 versus 0.818, respectively. In the high-specificity range of both tests, which is clinically the most relevant, the anti-CCP2 ELISA appears to be superior to the anti-MCV assay.

## Abbreviations

ACR = American College of Rheumatology; AKA = antikeratin antibody; anti-CCP = anti-cyclic citrullinated peptide; anti-MCV = anti-modified citrullinated vimentin; APF = antiperinuclear factors; AUC = area under the curve; CI = confidence interval; ELISA = enzyme-linked immunosorbent assay; Ig = immunoglobulin; IgM-RF = immunoglobulin M-rheumatoid factor; OA = osteoarthritis; PMR/GCA = polymyalgia rheumatica and/or giant cell arteritis; RA = rheumatoid arthritis; RF = rheumatoid factor; ROC = receiving operating characteristic; SD = standard deviation; SpA = spondyloarthritis.

## Competing interests

The authors declare that they have no competing interests.

## Authors' contributions

C Dejaco, C Duftner, MS, and MH diagnosed and treated the patients. MH, C Dejaco, HL, and C Duftner designed the study and performed the analyses. WK carried out the ELISAs. MS helped to coordinate the study. All authors were involved in drafting the manuscript and read and approved the final version.

## Supplementary Material

Additional File 1 A TIFF file showing a flow chart of enrolment and outcomes. Click here for file

## Figures and Tables

**Table 1:** Clinical characteristics of patients with rheumatoid arthritis (*n *= 164)

Age	60.4 (12.0)^a^
Female, *n *(%)	140 (85.4)
Disease duration (*n *= 153)	
<1 year, *n *(%)	23 (15.0)
1–5 years, *n *(%)	45 (29.4)
>5 years, *n *(%)	85 (55.6)
DAS-28 (*n *= 69)	3.3 (1.5)^a^
Swollen joint count (*n *= 137)	1 (0–23)^b^
Tender joint count (*n *= 92)	1 (0–28)^b^
Patients' global assessment (VAS) (*n *= 56)	23.2 (23.5)^a^
ESR (mm/first hour) (*n *= 152)	28.8 (19.3)^a^
CRP (mg/l) (*n *= 160)	13.9 (23.2)^a^
IL-6 (pg/ml) (*n *= 74)	111.0 (292.2)^a^

^a^Mean (standard deviation). ^b^Median (range). CRP, C-reactive protein (normal values 0–6 mg/l); DAS, disease activity score; ESR, erythrocyte sedimentation rate (normal values 0–10 mm/first hour); IL-6, interleukin-6 (normal values 0–3 pg/ml); VAS, visual analogue scale (range 0–100 mm).

**Table 2:** Comparison of sensitivity and specificity of the anti-modified citrullinated vimentin and the anti-cyclic citrullinated peptide test (*n *= 467)

	Cutoff (U/ml)	Sensitivity (% [95% CI])	Specificity (% [95% CI])
Anti-MCV	20.0^a^	69.5 (61.9–76.5)	90.8 (86.9–93.8)
	19.0^b^	70.1 (62.5–77.0)	89.8 (85.8–92.9)
	81.5^c^	53.7 (45.7–61.5)	98.7 (96.7–99.6)
Anti-CCP2	10.0^a^	70.1 (62.5–77.0)	98.7 (96.7–99.6)

^a^Recommended by the manufacturer. Cutoff with identical sensitivity^b ^and specificity^c ^compared with the anti-CCP2 test, respectively.

anti-CCP2, second-generation anti-cyclic citrullinated peptide; anti-MCV, anti-modified citrullinated vimentin; CI, confidence interval.
