# Supplementary figures and images for: Diagnostic value of antibodies against a modified citrullinated vimentin in rheumatoid arthritis
Source: Arthritis Res Ther. 2006 Jul 19;8(4):R119. doi: 10.1186/ar2008 (PMC1779400; doi:10.1186/ar2008)

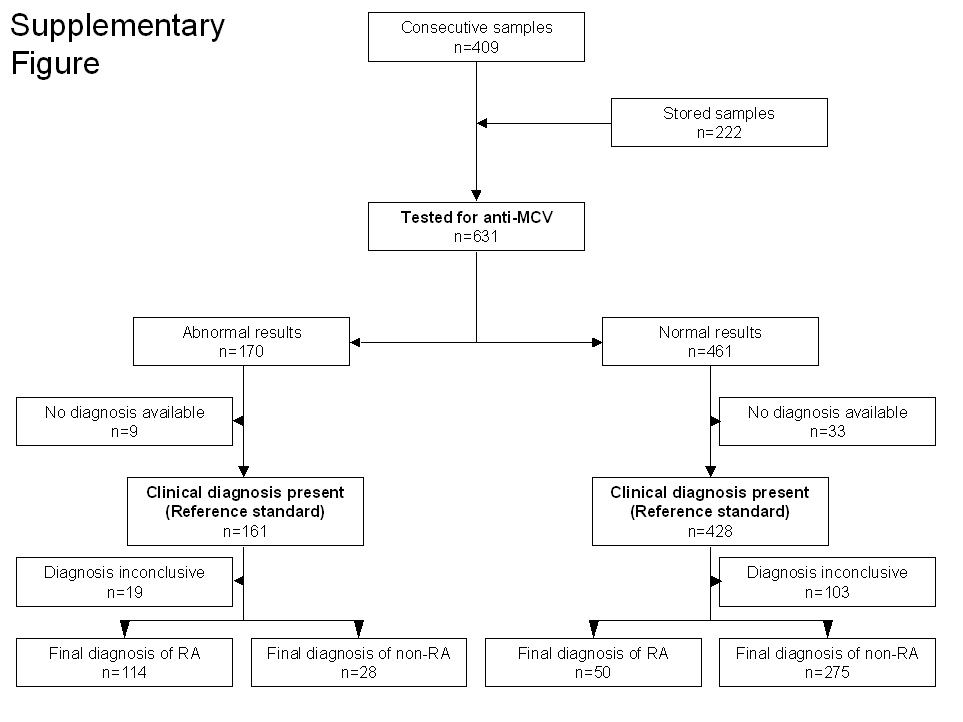

Supplement: Additional File 1 — A TIFF file showing a flow chart of enrolment and outcomes. [file ar2008-S1.tiff]
